# Reversed ageing of Fe_3_O_4_ nanoparticles by hydrogen plasma

**DOI:** 10.1038/srep20897

**Published:** 2016-02-23

**Authors:** Carolin Schmitz-Antoniak, Detlef Schmitz, Anne Warland, Nataliya Svechkina, Soma Salamon, Cinthia Piamonteze, Heiko Wende

**Affiliations:** 1 Peter-Grünberg-Institut (PGI-6), Forschungszentrum Jülich, 52425 Jülich, Germany; 2 Faculty of Physics and Center for Nanointegration Duisburg-Essen (CENIDE), Universität Duisburg-Essen, Lotharstr. 1, 47048 Duisburg, Germany; 3 Helmholtz-Zentrum Berlin für Materialien und Energie, Albert-Einstein-Str.15, 12489 Berlin, Germany; 4 Swiss Light Source, Paul Scherrer Institute, 5232 Villigen PSI, Switzerland

## Abstract

Magnetite (Fe_3_O_4_) nanoparticles suffer from severe ageing effects when exposed to air even when they are dispersed in a solvent limiting their applications. In this work, we show that this ageing can be fully reversed by a hydrogen plasma treatment. By x-ray absorption spectroscopy and its associated magnetic circular dichroism, the electronic structure and magnetic properties were studied before and after the plasma treatment and compared to results of freshly prepared magnetite nanoparticles. While aged magnetite nanoparticles exhibit a more *γ*-Fe_2_O_3_ like behaviour, the hydrogen plasma yields pure Fe_3_O_4_ nanoparticles. Monitoring the temperature dependence of the intra-atomic spin dipole contribution to the dichroic spectra gives evidence that the structural, electronic and magnetic properties of plasma treated magnetite nanoparticles can outperform the ones of the freshly prepared batch.

Magnetite nanoparticles are objects of intense research activities due to their broad range of applications covering technological, medical, and environmental applications. They are used e.g. for rotary shaft sealing, oscillation damping, position sensing^1^, magnetic inks for jet printing^2^, as contrast agents in magnetic resonance imaging^3^,^4^ and to remove heavy metals from wastewater^5^. In addition, magnetite is a half-metal with a predicted negative spin polarisation^6^,^7^ making magnetite interesting for spintronics^8^. For all applications, a high quality of magnetite is crucial to obtain the desired properties. But magnetite nanoparticles suffer from severe ageing effects when exposed to air even when they are dispersed in a purified solvent^9^,^10^,^11^. Here we show that this ageing can be fully reversed by a hydrogen plasma treatment. Characterisation by X-ray absorption spectroscopy gives evidence that the structural, electronic and magnetic properties of plasma treated magnetite nanoparticles can outperform the ones of the freshly prepared batch. In addition, the potential usage for nanoscale spintronic devices is discussed and it is suggested how to adopt the plasma cleaning procedure for particles that shall be used in aqueous solution.

Before presenting our results, some basic properties of magnetite shall be summarised. Magnetite is the Fe oxide with the highest net magnetic moment and crystallises in a cubic inverse spinel structure. In a simple picture, it consists of Fe^2+^ ions on octahedral lattice sites, Fe^3+^ on octahedral and Fe^3+^ on tetrahedral lattice sites. Although it is well-known that due to hybridisation effects the charges of the ions differ from the nominal values, we will use this notation here to distinguish between the different Fe species.

Pure magnetite shows a phase transition around 123 K, the so-called Verwey transition (VT)^12^, accompanied by a spin re-orientation transition at slightly higher temperature^13^ characterised by a vanishing magnetocrystalline anisotropy. In the low temperature phase, magnetite is an insulator, has a monoclinic structure connected to charge ordering^14^,^15^ and exhibits orbital order on the Fe^2+^ sites^16^,^17^. In addition it was found that localised electrons are shared between three Fe ions on octrahedral lattice sites which can be viewed as quasiparticles and were named *trimerons*^18^. Increasing deviation from the magnetite stoichiometry towards a higher degree of oxidation yields a significant lowering of the transition temperature^19^. Thus, the VT is a useful indicator for the quality of magnetite samples. However, at the nanoscale the VT is difficult to observe since diffraction methods suffer from severe peak broadening due to the small crystallite size. The occurence of superparamagnetism hampers the observation in magnetometry and measurements of the conductivity are difficult to interpret in the case of ensembles of nanoparticles, since simply the path of the electric current may change. The x-ray magnetic circular dichroism (XMCD) has recently been shown to be capable to omit these difficulties^20^. Therefore, we use the x-ray absorption spectroscopy, in particular analysis of the x-ray absorption near-edge structure (XANES) and its associated XMCD, to characterise the sample before and after hydrogen plasma treatment.

## Results

### Hydrogen plasma efficiency

**Figure 1:**
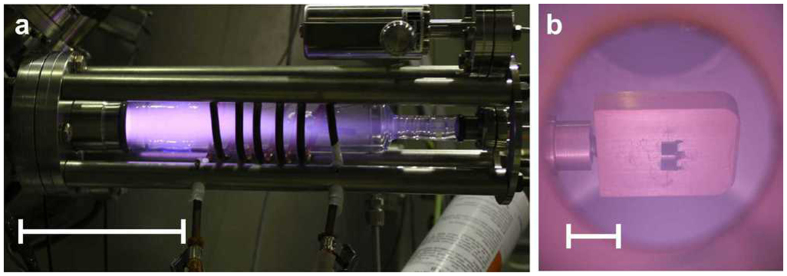
Hydrogen plasma. (**a**) Photograph of the plasma chamber with ignited hydrogen plasma at UE46-PGM1, HZB-BESSYII. Scale bar is 10 cm. (**b**) Photograph of two samples on a sample holder in the hydrogen plasma region. Scale bar is 1 cm. The samples are facing to the glass part of the plasma chamber, the photograph was taken through a window above.

A capacitive hydrogen plasma has already been used to reduce oxidised nanoparticles to the metallic state^21^. In this work, we try to end up with another oxide with a lower oxidation state. In general, during a plasma treatment, the sample may be contaminated by deposition of unintentionally sputtered material from the surrounding walls or from the electrode in the case of capacitively coupled plasma where the electrode is placed inside the UHV chamber. In that case, one has to take care to avoid large gradients of the electric field by a carefully rounded shape of the electrode. In this regard, the advantage of the inductively coupled plasma as used in this work is the absence of a strong electric field gradient inside the plasma chamber. The plasma chamber is shown in Fig. 1a. It consists of a glass tube surrounded by a copper coil. The power is fed into the plasma by electric currents produced by electromagnetic induction through a time-varying magnetic field. The latter is generated by the electric field component of a radio frequency electromagnetic wave through the copper coil. The plasma region can be seen by eye and does not only fill the glass part, but also the neighbouring preparation chamber where the sample is located (Fig. 1b). It cannot be excluded that some Si atoms are sputtered from the glass wall and deposited during the plasma treatment. However, we think that this is negligible due to the absence of large electric field gradients as mentioned above, the quite large distance of the sample from the main plasma zone and the generally low sputter efficiency of hydrogen. This is supported by Auger electron spectroscopy on a gold film that has been exposed for 2 hours to the hydrogen plasma where no contaminations could be observed.

**Figure 2:**
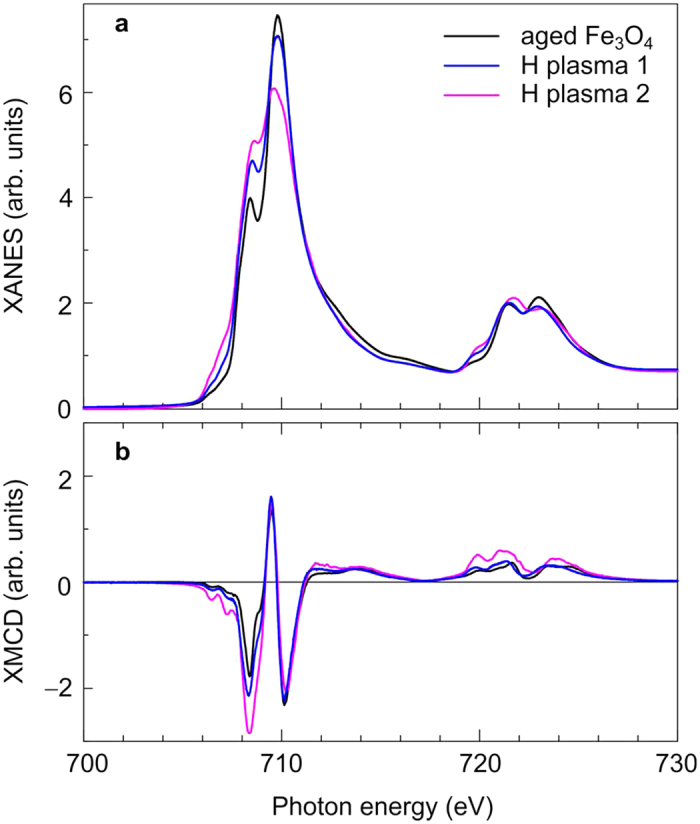
Plasma efficiency checked by x-ray absorption spectroscopy. X-ray absorption near-edge structure (**a**) and magnetic circular dichroism (**b**) at the Fe L_3,2_ absorption edges at room temperature for aged Fe_3_O_4_ nanoparticles (black line), Fe_3_O_4_ nanoparticles after incomplete hydrogen plasma treatment (blue line), and after a complete hydrogen plasma treatment (magenta line). Spectra were taken at *T* = 300 K in a magnetic field of 3 T.

A first hint of the hydrogen plasma efficiency are the spectral shapes of XANES and XMCD at the Fe L_3,2_ absorption edges as can be seen in Fig. 2a,b, respectively. Firstly, we will present the results from the the XANES analysis before turning to the results deduced from XMCD spectra. At the Fe L_3_ absorption edge around 710 eV, the double peak indicates Fe in an oxidic environment. A rule of thumb is: The higher the first peak with respect to the second one, the more Fe^2+^ ions are in the material with respect to Fe^3+^, i.e. the larger the magnetite fraction in our case. The black line shows the XANES of old magnetite nanoparticles. The particles were stored in a glove box under Ar atmosphere for about 17 months dispersed in purified ethanol. For the XANES/XMCD measurements, the particles were drop coated on a 5 mm × 5 mm large piece of a naturally oxidised p-doped Si wafer. By comparison with reference data from the literature^22^, it can be seen that the XANES is similar to the one of Fe_3−*δ*_O_4_ with *δ* = 0.23. After a first incomplete plasma treatment the spectral shape at the Fe L_3_ absorption edge has changed (blue line). The first peak grows at the expense of the second peak indicating more magnetite (*δ* < 0.13). This effect is even larger after a complete plasma treatment (magenta line). For the complete plasma treatment, the efficiency was enhanced by grounding the sample. In the latter case, the XANES is very similar to the one of pure magnetite (*δ* = 0)^22^. The XMCD at the Fe L_3_ absorption edge is W-shaped. The intensity of the first (negative) peak around 708.5 eV mainly refers to Fe^2+^ ions on octahedral lattice sites. A small fraction of the intensity is related to Fe^3+^ ions on octahedral lattice sites which have their main contribution to the XMCD at higher energies, i.e. the third (negative) peak at about 710 eV. The positive peak around 709.5 eV is due to Fe^3+^ ions on tetrahedral lattice sites, which are aligned antiparallel to the ions on octahedral sites leading to a reversed sign in XMCD. The growing first peak in the XMCD spectrum indicates the increasing amount of magnetically ordered Fe^2+^ ions upon hydrogen plasma treatment. In the following, we will denote the three peaks 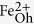, 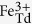, and 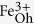, respectively, according to their nominal charge and lattice site, which is either octahedral (Oh) or tetrahedral (Td). One may note that this is an approximate approach, since the XMCD signals from different Fe species are energetically not well-separated. The interested reader can find the XMCD asymmetry spectra, i.e. the XMCD normalised to the total absorption, in the Supplementary Information.

### Quality of Fe_3_O_4_ nanoparticles

**Figure 3:**
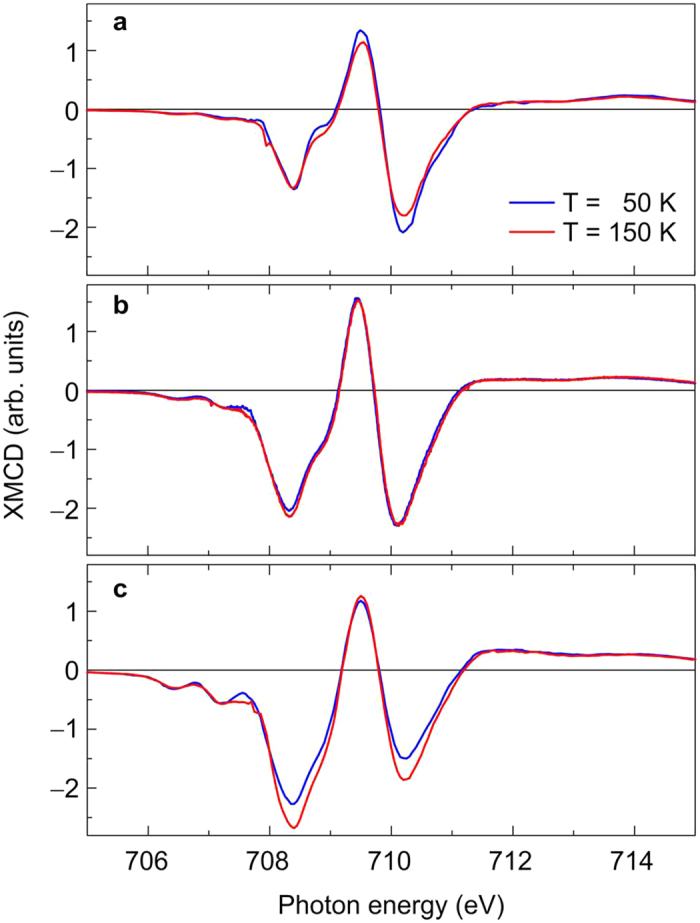
Temperature-dependent XMCD. X-ray magnetic circular dichroism for 50 K (blue lines) and 150 K (red lines) in a magnetic field of 3 T. Spectra were taken (**a**) before hydrogen plasma treatment, (**b**) after incomplete hydrogen plasma treatment, (**c**) after complete hydrogen plasma treatment.

As already mentioned in the introduction, measuring the temperature dependent XMCD is a sensitive tool to monitor the influence of the VT in magnetite that occurs in high-quality samples only. Analysing XANES and XMCD by an integral method, effective spin and orbital magnetic moments can be determined using the so-called sum rules^23^,^24^,^25^. The first is denoted *effective* spin magnetic moment 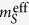, since it consists not only of the spin magnetic moment 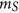, but an additional dipole term 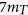 accounting for a possible asphericity of the spin density distribution. It has been shown that in the low temperature phase, 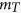 is sizeable for Fe^2+^ ions on octahedral sites while it can be neglected in the high-temperature cubic phase yielding a significant reduction of the effective spin magnetic moment at low temperatures related to the VT^20^. This can be already seen in the XMCD spectra. In Fig. 3 the XMCD is shown for two different temperatures, 50 K and 150 K. At 50 K magnetite is in its low-temperature phase, at 150 K in the high-temperature phase.

We focus on the behaviour of the XMCD at the Fe L_3_ absorption edge since the signal is larger and easier to interpret. In the case of the aged magnetite nanoparticles in panel (a), the XMCD is slightly larger at lower temperatures reflecting a usual temperature dependence of the magnetisation. Having a closer look at the fine structure, one may notice that this increase is mainly at the 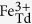 and 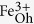 peaks observable. The first peak, 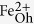, remains largely constant. After the first plasma treatment, the XMCD related to the Fe^3+^ ions is roughly the same at the two different temperatures while the 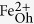 peak is slightly reduced at lower temperature indicating the phase transition (panel (b)). After the complete hydrogen plasma treatment, the reduction of the XMCD at lower temperature is obvious for both 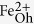 and 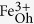, i.e. the two negative peaks related to Fe ions on octahedral lattice sites. This is a clear indicator for the VT that occurs only in magnetite of high quality.

**Figure 4:**
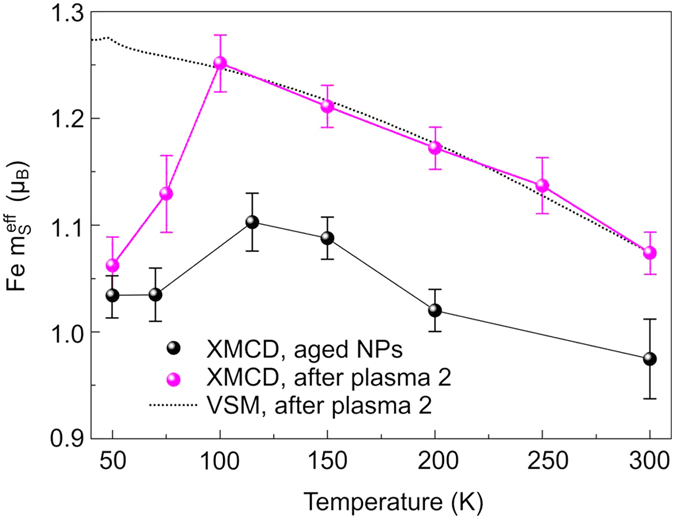
Temperature-dependent (effective) spin magnetic moments. Normalised effective spin magnetic moment of Fe as a function of temperature derived from x-ray magnetic circular dichroism (symbols) and scaled magnetisation measured by vibrating sample magnetometry (dotted line).

Figure 4 shows the temperature dependence of the effective spin magnetic moments calculated from XANES and XMCD for the aged magnetite nanoparticles (black symbols) and after the complete plasma treatment (magenta symbols). Details about the determination of magnetic moments can be found in the Supplementary Information. For comparison, magnetometry data obtained by a vibrating sample magnetometer (VSM) are shown. Apparently, the VSM is not sensitive to the spin dipole term^26^,^27^ that indicates the phase transition in magnetite.

## Discussion

For the aged nanoparticles a small kink is visible below 100 K (about 5% reduction) in the temperature-dependent effective spin magnetic moment 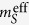 determined from XMCD. For freshly prepared magnetite this reduction is significantly larger (about 10% reduction^20^). In combination with the fact the the XANES of the aged nanoparticles corresponds to a more *γ*-Fe_2_O_3_-like electronic structure, this (i) confirms that the transition observed in XMCD is related to magnetite and (ii) suggests that there is still a magnetite core present in the aged nanoparticles.

After a complete plasma treatment, the transition of the nanoparticles is sharper and even more pronounced as for freshly prepared magnetite nanoparticles^20^ proving the high quality of magnetite after hydrogen plasma. This is essential for applications like e.g. in miniaturised spintronics since it has been shown that the half-metallicity with predicted negative spin polarisation^6^,^7^ is a property of pure magnetite. More precisely, a large room-temperature spin polarisation of −(80 ± 5)% near the Fermi energy of epitaxial Fe_3_O_4_(111) was found experimentally by photoelectrion spectroscopy^28^. In nanoparticles, besides the highly spin-polarised (111) surface, another stable surface of magnetite, i.e. the (001) surface, may be present. The (001) surface has the tendency to form an insulating surface reconstruction which lowers the spin polarisaion significantly^28^,^29^. In this regard, the hydrogen plasma treatment reveals a second advantageous property: atomic hydrogen is known to lift the surface bandgap and to recover the half-metallic character of the magnetite surface^29^,^30^.

For other applications of magnetite nanoparticles, like e.g. their use as contrast agent in magnetic resonance imaging, the high net magnetic moment is the decisive factor to favour magnetite over other Fe oxides like Fe_2_O_3_. In this case, the plasma treatment can be performed on the nanoparticles deposited onto NaCl that can be re-solved afterwards to obtain pure magnetite nanoparticles in aqueous solution.

In summary, the hydrogen plasma treatment turns out to be a useful tool to reverse the ageing effect of magnetite nanoparticles. The quality of the magnetite nanoparticles seems to be even higher than in the freshly prepared state as deduced from the observed transition in the XMCD. Since the presented hydrogen plasma treatment is quick and easy to apply, it is recommended as an additional preparation step when the main focus is on well-defined properties of nanoscale magnetite.

## Methods

### Nanoparticle synthesis

The nanoparticles were synthesised using a one-pot water-in-oil microemulsion technique as described elsewhere^31^. As summarised in^20^, FeCl_2_ and FeCl_3_ were used as precursors for the different Fe ions. IGEPAL (R) CO-520 as stabilising organic surfactant and ammonium hydroxide as catalyst. The mean diameter and its distribution was determined with transmission electron microscopy and found to be (6.3 ± 0.9) nm.

### X-ray absorption measurements

X-ray absorption measurements were carried out at the high-field end station at beamline UE46-PGM1, HZB-BESSY II, and X-Treme beamline^32^ at SLS. All spectra were taken in total electron yield (TEY) mode by measuring the sample drain current. The information depth is limited by the mean free path of the escaping electrons, i.e. about 2 nm. Since the nanoparticles have a mean radius of about 3 nm, the x-ray absorption spectra represent the average over the whole particle with a pronounced signal from the surface with respect to the core. For XMCD measurements, the sample was cooled down to the lowest temperature of about 5 K in a magnetic field of +0.5 T applied to avoid cooling in unknown remanence fields. Measurements were performed in a magnetic field of +3 T for different signs of circular polarisation of x-rays. Additionally, some spectra were taken with reversed magnetic field.

### Plasma treatment

The hydrogen plasma treatment was performed *in situ* after mounting the UHV compatible portable plasma chamber to the preparation chambers at the corresponding beamlines. The plasma was operated at a frequency of 13.59 MHz and a power of 20 W. No reflected power was obtained after the plasma was ignited and optimised using a suitable matchbox for radio frequencies. The hydrogen pressure was kept constant at (1.15 ± 0.05 × 10^-2^) mbar by controlling the gas flow through a leak valve manually.

## Additional Information

**How to cite this article**: Schmitz-Antoniak, C. *et al.* Reversed ageing of Fe_3_O_4_ nanoparticles by hydrogen plasma. *Sci. Rep.* **6**, 20897; doi: 10.1038/srep20897 (2016).

## Supplementary Material

Supplementary Information
