# Supplementary material for: Reversed ageing of Fe3O4 nanoparticles by hydrogen plasma
Source: Sci Rep. 2016 Feb 23;6:20897. doi: 10.1038/srep20897 (PMC4763261; doi:10.1038/srep20897)
Supplement: Supplementary Information [file srep20897-s1.pdf]

Reversed ageing of Fe_3O_4 nanoparticles by hydrogen plasma: Supplementary Information

Carolin Schmitz-Antoniak,[1,2,*] Detlef Schmitz,[3] Anne Warland,[2] Nataliya Svechkina,[1] Soma Salamon,[2] Cinthia Piamonteze,[4] and Heiko Wende[2]

[1] *Peter-Grünberg-Institut (PGI-6), Forschungszentrum Jülich, 52425 Jülich, Germany*

[2] *Faculty of Physics and Center for Nanointegration Duisburg-Essen (CENIDE), Universität Duisburg-Essen, Lotharstr. 1, 47048 Duisburg, Germany*

[3] *Helmholtz-Zentrum Berlin für Materialien und Energie, Albert-Einstein-Str.15, 12489 Berlin, Germany*

[4] *Swiss Light Source, Paul Scherrer Institute, 5232 Villigen PSI, Switzerland*

[*] Electronic address: `c.schmitz-antoniak@fz-juelich.de`

Supplementary methods

Sum rules based XMCD analysis. With XMCD analysis, effective spin and orbital magnetic moments of the unoccupied final states - in our case the 3d states of Fe - can be determined by using the so-called sum rules [23,24]. According to Chen et al. [25] the effective spin magnetic moment m_S^{eff} and orbital magnetic moment m_l divided by the number of unoccupied 3d states n_h is given by

$$\frac{m_S^{\text{eff}}}{n_h} = -\frac{3p - 2q}{r} \tag{S1}$$

$$\frac{m_l}{n_h} = -\frac{2q}{3r} \tag{S2}$$

with the integrals

$$p = \int_{L3} (\text{XMCD})\, \text{d}E \tag{S3}$$

$$q = \int_{L3+L2} (\text{XMCD})\, \text{d}E \tag{S4}$$

$$r = \int_{L3+L2} (\text{3d XANES})\, \text{d}E. \tag{S5}$$

Note that we define the XANES spectrum as the average between spectra with reversed photon helicity (or reversed magnetisation), $(\mu_+ + \mu_-)/2$. Chen et al. [25] use the sum $(\mu_+ + \mu_-)$ resulting in an additional factor of 2 in equations S1 and S2. The value r is the integral of the XANES connected to electron transitions from the $2p_{3/2}$ and $2p_{1/2}$ into the 3d states. To separate these transitions from transitions into higher states, the latter are described by two step-like functions, at the L_3 threshold energy and the L_2 energy respectively. The threshold energies $E(L_3)$ and $E(L_2)$ are determined by the maximum slope of the absorption. The step height at the L_3 absorption edge is twice as large as for the L_2 edge, since there are twice as many electrons in the initial $2p_{3/2}$ states compared to the $2p_{1/2}$ states. After normalisation of the XANES to unity in the post-edge region, the transitions into higher electronic states than 3d are thus estimated by

$$\frac{2}{3}\left(\exp\left[E(L_3) - E\right] + 1\right)^{-1} + \frac{1}{3}\left(\exp\left[E(L_2) - E\right] + 1\right)^{-1} \tag{S6}$$

After subtraction of this function from the normalised XANES, the resulting spectrum is integrated to determine the value of r in the sum rules. XANES, step-like functions and integrals of 3d XANES are shown in Fig. S1 for the aged magnetite sample (a) and after

complete hydrogen plasma treatment (c). Low-temperature spectra are shown in blue, high-temperature spectra in red. Due to the strong oscillatory behaviour of the XANES, a high energy was chosen for normalisation and determination of r, i.e. 771 eV.

For the aged magnetite only very small changes in the XANES spectra at low temperatures compared to high temperatures are visible in particular at the first peak of the L_3 absorption edge. The integral value remains largely constant. After complete hydrogen plasma treatment, the XANES and the 3d XANES integral is obviously smaller (about 5...6%) at low temperatures compared to high temperatures. The similar shape of the 3d XANES integral indicates that this is not caused by inadequate normalisation. The absence of the temperature dependent change in intensity in the case of the aged nanoparticles shows that this is not an experimental artefact or technical problem. Thus, one may already conclude that the reduced 3d XANES integral at temperatures below 100 K is a property of magnetite nanoparticles.

The number of unoccupied d states n_h is roughly proportional to the 3d XANES intensity. Assuming $n_h = 4.67$ for magnetite in the high-temperature phase according to the average of Fe^{2+} ($3d^6$, $n_h = 4$) and two Fe^{3+} ($3d^5$, $n_h = 5$), for the case of the aged nanoparticles we found by comparison of the 3d XANES integrals a value of $n_h \approx 5.0$ indicating a further oxidation. In the low temperature phase of magnetite we found $n_h \approx 4.3$. The reduced number of unoccupied d states at low temperatures with respect to high temperatures may indicate a reduced electron transfer to the oxygen atoms, i.e. less hybridisation in agreement to the model of electron localisation below the Verwey transition temperature.

As some people prefer to choose the step-like functions in a way that they do not cross the spectra, we repeated the analyses with the two step-like functions scaled by 0.69 and choose the upper limit of the integral at $E = 731$ eV. No significant differences were found in the calculated magnetic moments.

XMCD spectra and their integrals are presented in Fig. S1 for the aged sample (b) and after complete hydrogen plasma treatment (d). In all cases, it is obvious that the orbital magnetic moment determined from the integral of the XMCD over both L_3 and L_2 absorption edges is negligibly small since the integral oscillates around zero at high energies. These oscillations are known as magnetic extended x-ray absorption fine structure (MEXAFS) [S2, S3] and were already dicussed for the case of magnetite [S1]. For the calculation of the orbital and effective spin magnetic moments, the values of q and p were

taken at $E = 767\,\text{eV}$ and at $E = 718\,\text{eV}$, respectively. For the number of unoccupied d states, we used $n_h = 5$ for the aged nanoparticles, $n_h = 4.67$ for the plasma treated nanoparticles in the high-temperature phase, and $n_h = 4.3$ in the low temperature phase of plasma treated particles as explained above. The temperature dependence of the effective spin magnetic moment is shown in Fig. 4 of the main manuscript. Note that the drop of the effective spin magnetic moment at low temperatures is also clearly visible when using the same n_h for both high and low temperatures. The change of the effective spin magnetic moment can already be seen in the XMCD integral which is smaller around the absorption edges at low temperatures. However, since the 3d XANES intensity is also reduced, this may lead to a misimpression. To avoid this, plotting the XMCD asymmetry instead of the XMCD shall be favoured. The XMCD asymmetry is the difference between the absorption spectra for reversed photon helicity (or reversed magnetisation) divided by the sum of the two spectra. In addition to the XMCD spectra in Fig. 3, we present the XMCD asymmetry in Fig. S2. Qualitatively, it shows the same temperature dependence as the XMCD spectra, i.e. the absolute values of the asymmetry increase from high to low temperature, because the effective spin magnetic moment increases according to a Bloch law (Fig. S2a) and decreases because the negative intra-atomic dipole moment of Fe^{2+} ions on octahedral sites reduces the effective spin magnetic moment below the Verwey transition [20].

Spin-orbit sum rules based XANES analysis.

At first glance, the vanishing orbital magnetic moment calculated from XMCD is in disagreement to the finding that the measurable intra-atomic dipole term is connected to the large spin-orbit coupling [20]. The ostensible contradiction shall be discussed here in more detail. Due to the integral method, effective spin and orbital magnetic moments determined by the XMCD sum rules are average values of all the different Fe ions which are expected to partly compensate each other due to the ferrimagnetic order. A more suitable way to estimate the orbital magnetism in this case is application of the so-called spin-orbit sum rules to the XANES [S4, S5]. These sum rules connect the expectation value of the angular part of the 3d spin-orbit coupling (SOC) with the branching ratio at the $L_{3,2}$ absorption edges. The branching ratio B is given by the integral of the 3d XANES at the L_3 absorption line divided by the integral over both L_3 and L_2 absorption edges

$$B = \frac{\int_{L3}(\mathrm{3d\,XANES})\,\mathrm{d}E}{\int_{L3+L2}(\mathrm{3d\,XANES})\,\mathrm{d}E}. \tag{S7}$$

No change in the branching ratio with temperature was found and it remains largely unchanged for the aged and the hydrogen plasma treated nanoparticles. Depending on the chosen height of the two step-like functions, the branching ratio for the 3d transition is between 0.8 and 0.85. This is significantly higher than the statistical branching ratio of 2/3. It is connected to the angular part of the 3d SOC $\langle l \cdot s \rangle$ according to [S6]

$$B = B_0 + (B_0 - 1)\,\frac{\langle l \cdot s \rangle}{n_h} \tag{S8}$$
$$\Leftrightarrow \frac{\langle l \cdot s \rangle}{n_h} = \frac{B - B_0}{B_0 - 1}$$

with the coefficient B_0 taking into account the electrostatic interaction between 2p and 3d electrons. If there is no 2p - 3d electrostatic interaction, the value of B_0 equals the statistical branching ratio 2/3. For $3d^6$ and $3d^5$ configuration, B_0 is around 0.725 and 0.75, respectively, according to Fig. 6 in Ref. [S5]. Thus, we assume an averaged value of $B_0 = 0.74$ and obtain $\langle l \cdot s \rangle / n_h \approx -0.26$ for $B = 0.8$ (reduced height of two step-like function) and $\langle l \cdot s \rangle / n_h \approx -0.42$ for $B = 0.85$ (height of two step-like function as shown in Fig. S1) in agreement to $\langle l \cdot s \rangle / n_h = -0.4 \pm 0.05$ in Ref. [S7]. This sizeable value of the expectation value of the angular part of the 3d SOC indicates non-vanishing orbital moments at the different Fe ions that compensate each other resulting in a negligible averaged orbital magnetic moment as determined by XMCD. Due to the SOC, the intra-atomic dipole term contributing to the effective spin magnetic moment does not average out even for randomly oriented crystallographic axes in an ensemble of nanoparticles. In a simple picture, the spin density distribution (partly) aligns with respect to an external magnetic field, yielding a non-vanishing contribution of the intra-atomic magnetic dipole term for all possible crystallographic orientations.

Assuming a similar relation between the SOC $\langle l \cdot s \rangle / n_h$ and the ratio of orbital-to-spin magnetic moment as in Ni [S6], the orbital magnetic moment estimated from the branching ratio of the XANES is between $m_l \approx 0.33$ ($B = 0.8$) and $m_l \approx 0.56$ ($B = 0.85$). This huge absolute value of the orbital magnetic moment is approximately the same for octahedral and tetrahedral lattice sites, but compensates each other. Thus, a non-vanishing orbital magnetic moment has to be present also at the $\mathrm{Fe}^{3+}_{\mathrm{Td}}$ ions indicating the breakdown of the

simple picture of a pure 3+ valency in agreement to Ref. [S7].

[S1] Goering, E., Gold, S., Lafkioti, M. & Schütz G. Vanishing Fe 3d orbital moments in single-crystalline magnetite. *Europhys. Lett.* **73**, 97–103 (2006).

[S2] Wende, H. Recent advances in x-ray absorption spectroscopy. *Rep. Prog. Phys.* **67**, 2105–2181 (2014).

[S3] Schütz, G. et al. Spin-dependent extended x-ray-absorption fine structure: Probing magnetic short-range order. *Phys. Rev. Lett.* **62**, 26202623 (1989).

[S4] van der Laan, G. & Thole, B. T. Local Probe for Spin-Orbit Interaction. *Phys. Rev. Lett.* **60**, 1977–1980 (1988).

[S5] Thole, B. T., van der Laan, G. Branching ratio in x-ray absorption spectroscopy. *Phys. Rev. B* **38**, 3158–3171 (1988).

[S6] Dhesi, S. S. et al. Electronic and magnetic structure of thin Ni films on Co/Cu(001). *Phys. Rev. B* **60**, 12852–12860 (1999).

[S7] Goering, E. Large hidden orbital moments in magnetite. *Phys. Status Solidi B* **248**, 2345–2351 (2011).

Supplementary figures

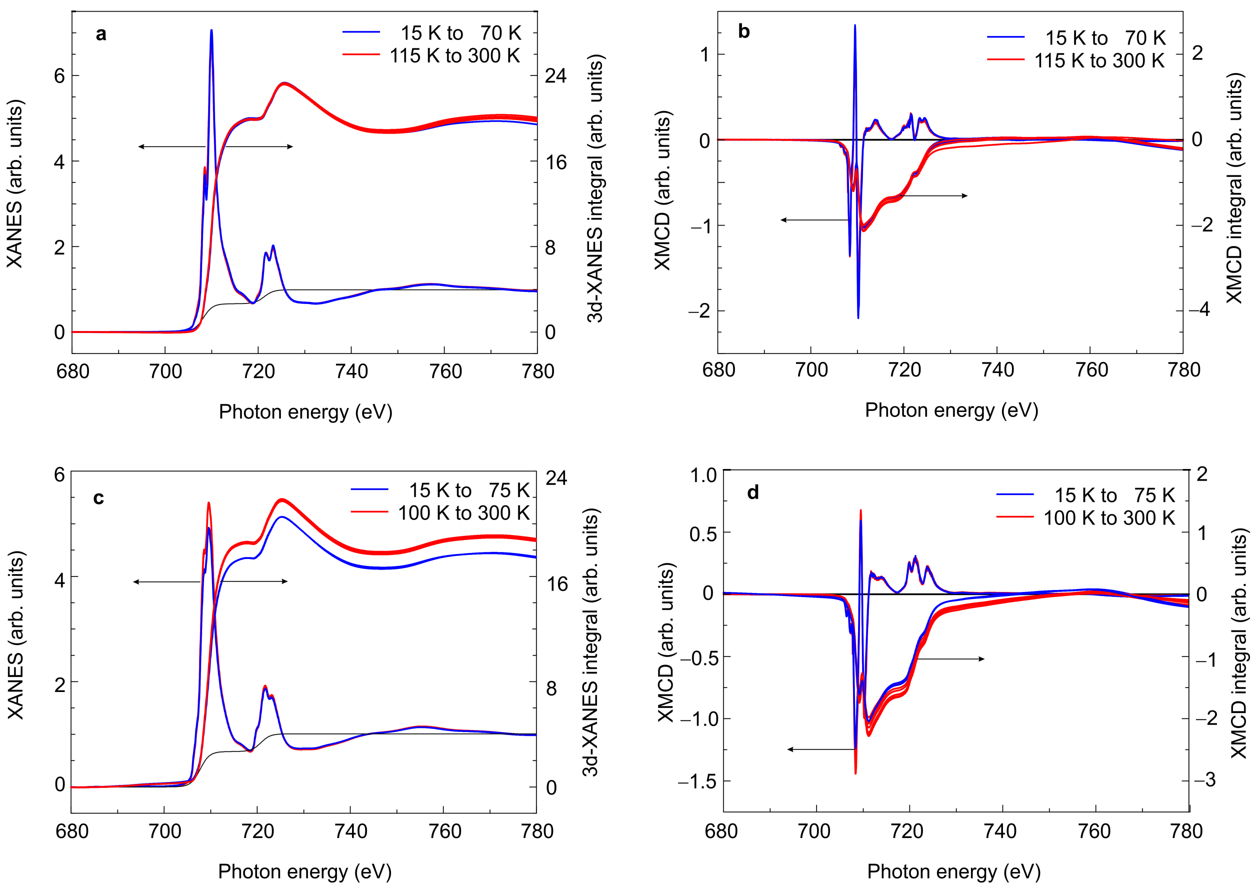

Supplementary Figure S1: **XANES, XMCD and their integrals.** a: XANES and integral of 3d XANES for aged magnetite nanoparticles. b: XMCD and its integral for aged magnetite nanoparticles. c: XANES and integral of 3d XANES for magnetite nanoparticles after complete hydrogen plasma treatment. b: XMCD and its integral for magnetite nanoparticles after complete hydrogen plasma treatment. a–d: Low-temperature spectra are shown in blue, high-temperature spectra in red. a,c: Black line represents absorption due to electron transitions into higher than 3d states.

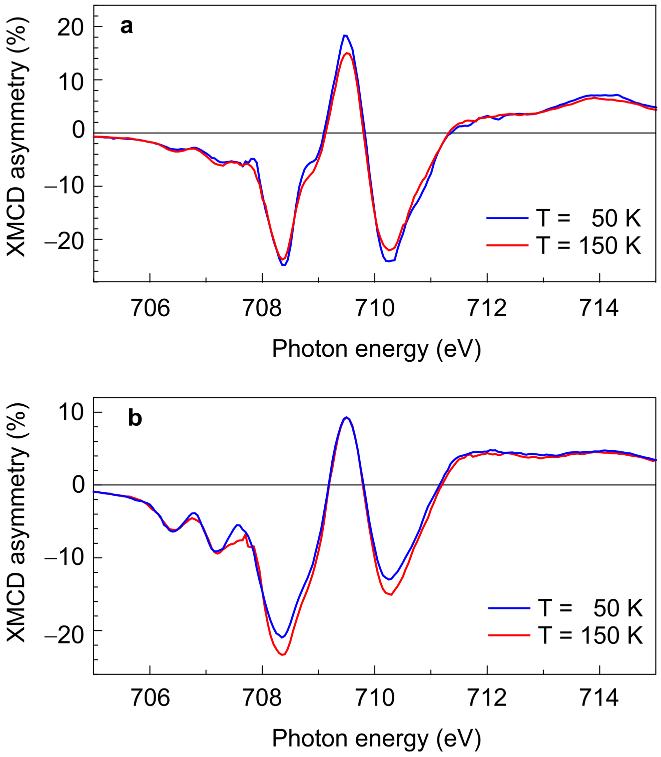

Supplementary Figure S2: **XMCD asymmetries at 150 K and 50 K.** X-ray magnetic circular dichroism asymmetry for 50 K (blue lines) and 150 K (red lines) in a magnetic field of 3 T. Spectra were taken a: before hydrogen plasma treatment, b: after complete hydrogen plasma treatment.
